# Co-Blend Application Mode of Bulk Fill Composite Resin

**DOI:** 10.3390/ma12162504

**Published:** 2019-08-07

**Authors:** Mohammad Al-Nabulsi, Alaa Daud, Cynthia Yiu, Hanan Omar, Salvatore Sauro, Amr Fawzy, Umer Daood

**Affiliations:** 1 Restorative Dentistry Specialist, Primary Health Care Corporation, Ministry of Health, Doha 26555, Qatar; 2 Restorative Department, School of Oral & Dental Sciences, University of Bristol Dental School & Hospital, Bristol BS8 1TH, UK; 3 Paediatric Dentistry and Orthodontics, Faculty of Dentistry, The University of Hong Kong, Hong Kong 99907, China; 4 Missouri school of dentistry and oral health (MOSDOH)—ATSU, Kirksville, MO 63501, USA; 5 Dental Biomaterials and Minimally Invasive Dentistry Departmento de Odontologia Facultad de Ciencias de la Salud Universidad, CEU-Cardenal Herrera, 46920 Valencia, Spain; 6 UWA Dental School, University of Western Australia, Nedlands, WA 6009, Australia; 7 Clinical Dentistry, Restorative Division, Faculty of Dentistry, International Medical University Kuala Lumpur, 126, Jalan Jalil Perkasa 19, Bukit Jalil, Bukit Jalil, Wilayah Persekutuan Kuala Lumpur 57000, Malaysia

**Keywords:** co-blend, resin, dentine, bulk-fill, bond strength, Raman, nanoindentation

## Abstract

**Objective:** To evaluate the effect of a new application method of bulk-fill flowable composite resin material on bond-strength, nanoleakage, and mechanical properties of dentine bonding agents. Materials and methods: Sound extracted human molars were randomly divided into: manufacturer’s instructions (MI), manual blend 2 mm (MB2), and manual blend 4 mm (MB4). Occlusal enamel was removed and flattened, dentin surfaces were bonded by Prime & Bond universal (Dentsply and Optibond FL, Kerr). For the MI group, adhesives were applied following the manufacturer’s instructions then light-cured. For MB groups, SDR flow+ bulk-fill flowable composite resin was applied in 2- or 4-mm increment then manually rubbed by a micro brush for 15 s with uncured dentine bonding agents and the mixture was light-cured. Composite buildup was fabricated incrementally using Ceram.X One, Dentsply nanohybrid composite resin restorative material. After 24-h water storage, the teeth were sectioned to obtain beams of about 0.8 mm^2^ for 24-h and thermocycled micro-tensile bond strength at 0.5 mm/min crosshead speed. Degree of conversion was evaluated with micro-Raman spectroscopy. Contraction gaps at 24 h after polymerization were evaluated and atomic force microscopy (AFM) nano-indentation processes were undertaken for measuring the hardness across the interface. Depth of resin penetration was studied using a scanning electron microscope (SEM). Bond strength data was expressed using two-way ANOVA followed by Tukey’s test. Nanoindentation hardness was separately analyzed using one-way ANOVA. Results: Factors “storage F = 6.3” and “application F = 30.11” significantly affected the bond strength to dentine. For Optibond FL, no significant difference in nanoleakage was found in MI/MB4 groups between baseline and aged specimens; significant difference in nanoleakage score was observed in MB2 groups. Confocal microscopy analysis showed MB2 Optibond FL and Prime & Bond universal specimens diffusing within the dentine. Contraction gap was significantly reduced in MB2 specimens in both adhesive systems. Degree of conversion (DC) of the MB2 specimens were numerically more compared to MS1 in both adhesive systems. Conclusion: Present study suggests that the new co-blend technique might have a positive effect on bond strengths of etch-and-rinse adhesives to dentine.

## 1. Introduction

Resin-based composite restorative materials are a cornerstone in modern-day dental practice because of their excellent esthetics and chemo-mechanical properties [1,2,3]. Dentine hybridization through polymeric agents represents a considerable innovation in dentistry, which has become clinically feasible [4]. New bonding strategies have emerged to improve and facilitate resin–dentine bonding [5]. Although almost all of the products currently in the market can achieve acceptable immediate bond strength, adhesive bonds degrade over a period of time [6]. Solutions to this have always remained elusive, as contributing mechanisms and the degree of degradation analyzed has never been consistent for all adhesive systems [7,8]. Acid-etching (35–37% phosphoric acid) procedure improves the performance of composite resin restorations when placed on enamel, but [9] this does not work as well with dentine [10] because of its heterogeneity in structure and composition representing a complex biological substrate [11,12]. The use of hydrophilic resin monomers to penetrate acid-etched demineralized dentine have improved the immediate performance of resin–dentine bonding systems [4]; but is not the same in terms of performance for long-term periods.

Manufacturers have simplified the adhesive procedures by making modern bonding agents which are more user-friendly; two-step etch-and-rinse, one-step self-etch and universal adhesives are the current simplified adhesive systems available in dental clinics [13]. This category of adhesive creates a semi-permeable [14,15,16] resin–dentine interface that permits dentinal fluid movement across the polymerized adhesives [17,18]. Furthermore, high water content of these adhesive and the” in-optimum” penetration of resin monomer through demineralized dentine leave residual water in the inter-fibrillar spaces, which activate host-derived protease enzymes (i.e., matrix metalloproteinases and cysteine cathepsin) [19] reducing the durability of dentine bonded interface [20]. Excessive water within the hybrid layer can prevent optimal polymerization of adhesive resin monomers [21], causing phase separation and plasticization of resin adhesive over a period of time [22].

From the literature, improvements in bond strength and reduction in nanoleakage of simplified adhesive systems can be achieved by ethanol wet-bonding technique [23,24], multiple application of adhesive layers [25,26], applying a hydrophobic extra bonding layer [27,28,29], co-cure [30] and sonic applications of the bonding agents [31,32]. Nevertheless, there still exists some controversy as to which technique is considered as the best bond and has the ability to seal dentinal tissue. In this study, a new application technique for bulk-full flowable composite resin with dentine bonding agent termed as the “manual blend technique” [MB] will be evaluated. This laboratory study aims at examining the degree of conversion through micro-Raman spectroscopy of adhesives applied with bulk-fill flowable composite. Shrinkage gaps (CG) at 24 h were evaluated for both the control and MB groups. AFM nano-indentation was used to analyze hardness (Hi) across the composite–dentine interface. Moreover, it was also analyzed, whether the manual blend technique can improve the resin–dentine bond strength and reduce nanoleakage at immediate and after thermocycling aging. The null hypotheses tested were that new application method [MB] of bulk-fill flowable composite resin materials with dentine bonding agents (i) has no effect on the micro-tensile bond strengths at 24 h or after ageing, (ii) has no effect on the nanoleakage and degree of polymerization (iii) has no effect on the contraction gaps after polymerization.

## 2. Materials and Methods

### 2.1. Teeth Selection and Preparation

**Figure 1:**
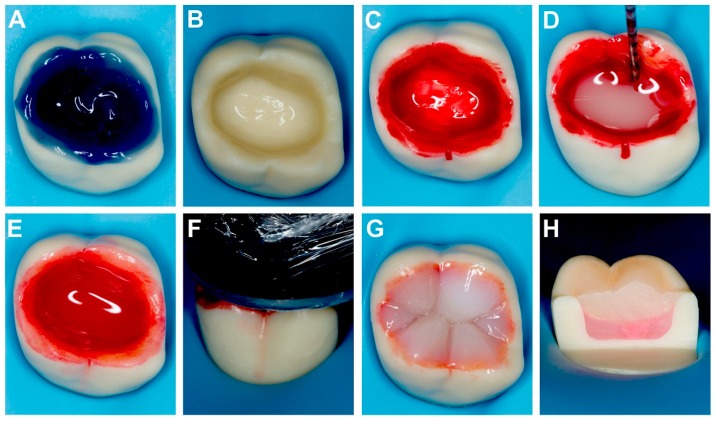
Adhesive procedure for the MB2 group; 2 mm of bulk-fill flowable materials rubbed over uncured dentine bonding agent using sterilized micro brush on dentine for 15 s, and light-cured for 20 s. (**A**) Phosphoric acid applied for 30 s on enamel and 15 s on dentine; (**B**) excess water removed without desiccation and (**C**) dentine bonding agent applied as per manufacturer’s instructions (red die added); (**D**,**E**) bulk-fill flowable composite resin manually applied (measured with probe) by micro brush with procured dentine bonding agent on dentine and rubbed for 15 s; (**F**) the mixture is light-cured by LED curing unit (600 mW/cm^2^) for 20 s; (**G**) incremental layering technique to build occlusal morphology in the following sequence—mesiopalatal, distobuccal, mesiobuccal, and distopalatal cusps; (**H**) sagittal cut made in mesiodistal directions showing 2 mm reddish layer of dentine bonding agent with bulk flowable composite resin covered by nanohybrid composite resin.

A total of 42 sound human third molars were used in the current study. Teeth were extracted from patients (age range 21–34 years), stored in 0.4% chloramine T solution at 4 °C for no more than three months. Patient’s informed consent was obtained to collect the extracted teeth under a protocol approved by the Institutional Review Board (Grant number: IMU 423/2018). Teeth were randomly divided into three groups (*n* = 14); manufacturer’s instructions (MI), manual blend 2 mm (MB2), and manual blend 4 mm (MB4). A slow-speed diamond-impregnated disc (Isomet, Buehler Ltd., Lake Bluff, IL, USA) was used under running water to create a flat mid-coronal dentine surface (1 mm below the dentine-enamel junction) by cutting occlusal enamel surface perpendicular to the longitudinal axis of each tooth. To standardize the smear layer, the exposed dentine surface was polished with 600-grit wet silicon carbide paper, and the samples ultrasonically rinsed in deionized water for 5 min. For placement of commercial bulk fill composite systems, the required increment depth was measured by a periodontal probe for the accuracy before polymerization (Figure 1). 

### 2.2. Microtensile Bond Strength

Teeth were prepared for adhesive procedure by acid-etching (32% phosphoric acid, Uni-Etch, Bisco Inc., Schaumburg, IL, USA) on exposed dentine surfaces for 15 s, and rinsed with deionized water (15 s) and kept visibly moist. Two groups (*n* = 5/group) were randomly created from teeth to test Prime & Bond Universal (PB) (Dentsply Sirona—Dentsply Caulk, Milford, DE, USA) and Optibond FL (OF; Kerr Corporation, Orange, CA, USA) adhesives. The compositions of the two adhesives and restorative composites materials are shown in Table 1 and Table 2, respectively. 

For MI group, two consecutive layers of adhesive (fully saturated micro-brush applicator) were individually applied to the wet demineralized dentine surface for 15 s, and gently air-dried for 5 s to evaporate the solvent. A light-emitting diode curing unit (Elipar S10, 3M ESPE, Minneapolis, MN, USA) with an output intensity of 600 mW/cm^2^ was used to light-cure the adhesive at room temperature. A light-curing nanohybrid composite resin (Ceram.X One Dentsply Sirona—Minneapolis, MN, USA) was applied to create 4 mm build-ups in 1 mm thickness increments which were individually light-cured for 20 s.

For the MB2 group, 2 mm of bulk-fill flowable composite resin material (Surefil SDR Flow+, Dentsply—Caulk, Berlin Germany) was placed above the uncured dentine bonding agent, manually rubbed using sterilized micro brush on dentine for 15 s, and light-cured for 20 s. For the MB4 group, 4 mm of bulk-fill flowable composite resin material was placed above the uncured resin, activated and cured the same way as described previously. Composite resin buildups were performed with a light-cured Ceram.X One (1281: DeTrey, Dentsply, Berlin, Germany) in two 1-mm thick increments each light-cured individually for 20 s. The specimens were stored in distilled water at 37 °C for 24 h. After 24 h, specimens were sectioned occluso-gingivally into 0.9 mm × 0.9 mm composite-dentine beams and half of the specimens were randomly divided and assigned for either immediate microtensile bond strength (μTBS) evaluation or thermocycling (10,000 cycles at temperatures ranging 5 °C to 55 °C). Using a cyanoacrylate adhesive (Zapit, Dental Ventures of North America, Corona, CA, USA), every single beam was attached to a Bencor Multi-T device (Danville Engineering, San Ramon, CA, USA) at each time point and the beams were stressed to failure under tension using a universal testing machine (Model 4440, Instron, Inc., Canton, MA, USA) at a crosshead speed of 1 mm/min. Using digital calipers (Model CD-6BS; Mitutoyo, Tokyo, Japan), the site of fracture was measured to the nearest 0.01 mm of the cross-sectional area for each specimen. Microtensile bond strength values were presented in MPa describing the force at debonding/cross-sectional surface area. The dentine side of the fractured beams were mounted on aluminium stubs to determine the mode of failure. The specimens were sputter-coated with gold/palladium and examined under a scanning electron microscope (SEM; Hitachi High Technologies America, Hitachi S-3400N, Inc., Schaumburg, IL, USA) operating at 15 kV. Beams were classified according to the modes of failure: (i) If the fracture site was within the adhesive, then it is adhesive failure, (ii) if the fracture site extended into either the dentine or the resin composite, then it is a mixed failure, (iii) cohesive failure in resin composite, and (iv) cohesive failure in dentine. 

### 2.3. Nanoleakage Analysis 

For the purpose of interfacial nanoleakage evaluation, 24 teeth (*n* = 4/group) were subjected to the same bonding procedures as described above, stored in artificial saliva at 37 °C for evaluation at 24 h and thermocycling regimen described above. Two coats of fast-drying nail varnish were applied 1 mm far from the bonded interfaces for specimens of different time points, followed by immersing the specimens in 50 wt.% ammoniacal silver nitrate solution (pH of 9.5). Specimens were later transferred to a photo developing solution (Kodak Professional Dektol Developer, Rochester, New York, USA) for 8 h after being rinsed and washed with deionized water for 5 min. The reduction of diamine silver ions into metallic silver grains was accomplished under fluorescent light [33]. Specimens were eventually removed from the solution and polished with diamond pastes (6 μm, 3 μm, 1 μm; Buehler Ltd., Lake Bluff, IL, USA) using a polishing cloth. Subsequently, an ultrasonic cleaner (T-1449-D, SP, Brazil) was used to carefully clean and remove the nail varnish for 10 min, air-dried and mounted on aluminum stubs. Finally, the specimens were stored in a desiccator for 24 h. The specimens were carbon coated and examined under a SEM in a back-scattered mode (15 kV), focusing on the silver tracer expression along the resin-dentine interface. A total of 40 images of the resin-dentine interfaces (500×) were obtained from each group. Two examiners evaluated the extent of silver deposition along the interface applying the classification method [34], to describe the extent of silver deposition along the interface: 0, indicating no nanoleakage; (1), if <25% nanoleakage; (2), 25–50% nanoleakage; (3), 50–75% nanoleakage, and (4) >75% nanoleakage.

### 2.4. Shrinkage and Gap Evaluation

Occlusal enamel was removed perpendicular to the long axis of the teeth (*n* = 12) (Isomet, Buehler Ltd., Lake Bluff, IL, USA) under water cooling. The exposed dentine surfaces were polished using 180-grit silicon carbide paper under continuous water irrigation. The resin-dentine specimens were prepared for groups as mentioned previously. Each specimen (2 × 2 mm) was immediately cross sectioned with a slow speed saw and polished for light microscopy evaluation (Image Analyzer, Olympus Stream, MPLAPON Plan Apochromat objective lens). Average of three gaps width measurements at randomly selected positions along the 70 µm interface width at the center were recorded as gap measurements. The location was classified as (1) interfacial, if the separation occurred at more than 50% of the observed interface, (2) cohesive, if it was in adhesive or dentin, and (3) ‘mixed’ if the fracture was both interfacial and cohesive.

### 2.5. AFM Nanoindentation

Resin–dentine slabs were prepared as mentioned previously for each group (*n* = 4). The specimens were cut perpendicularly to the bonding area (Isomet, Buehler Ltd., Lake Bluff, IL, USA) under water cooling using a diamond saw to obtain three resin–dentine slabs with a thickness of 1.5 mm. The slabs were polished using #800-grit SiC abrasive papers (Struers LaboPol-4), followed by diamond pastes (Buheler-MetaDi, Buheler Ltd. Lake Bluff, IL, USA) through 1 µm. The specimens were finally immersed in ultrasonic bath (QS3, Ultrawave Ltd., Cardiff, UK) containing deionized water for 10 min. As the specimens were stored in deionized water (pH 7.0), nano-hardness was analyzed in water immersion using an indentor system (Hysitron Inc., Minneapolis, MN, USA) and atomic force microscopy (AFM Nanoscope V, Digital Instruments, Veeco Metrology group, Santa Barbara, CA, USA) equipped with a Berkovich diamond cube corner indenter with a tip radius of approximately 20 nm. Five indentations were performed in a straight line with a load of 4000 nN and a time function of 10 s suitable for a standardized evaluation. This was taken into consideration for each specimen with one indentation in the middle of the hybrid layer (HL) and down to the intertubular dentine. The distance between each indentation was standardized by adjusting the load function and distance intervals in 5 (±1) µm steps [35]. 

### 2.6. SEM Resin–Dentine Interface

A total of 24 teeth were used for analysis of the resin–dentine interface of specimens prepared as previously mentioned in the above test sessions. Specimens (*n* = 2) were sectioned perpendicular to the bonded surface exposing the adhesive interface obtaining resin–dentine slabs (1 mm thick). As two slabs were obtained from each tooth, the slabs were wet-polished using #800-grit SiC papers (Carbimet, Buehler, Lake Bluff, IL, USA) and rinsed thoroughly using distilled water for 10 min. The specimens were later subjected to ultrasonic agitation and dried using absorbent paper (Kimwipes; Kimberly-Clark Professional, Roswell, GA, USA). To analyze the resin tags, resin–dentine slabs were etched using 35% phosphoric acid gel (15 s), rinsed with distilled water (15 s), air dried, and then submerged in 5.25% sodium hypochlorite solution (20 min). Specimens were later washed with distilled water for 5 min and sequentially dried using ascending grades of 50%, 75%, 80%, 95%, and 100% ethanol. Specimens were transferred to critical point dryer (Balzers 030, Shimadzu, Kyoto, Japan) for desiccation. All specimens were mounted on aluminum stubs using conductive tape (double-sided carbon tape) and then gold sputtered for 120 s (Baltec SCD sputter, Scotia, NY, USA). After gold sputtering, the specimens were viewed using SEM (Philips/FEI XL30 FEG SEM, Hillsboro, OR, USA), operated at an accelerating voltage of 10 kV at different magnifications.

### 2.7. Degree of Polymerization and Raman Data Acquisition/Processing

The degree of conversion (DC) of adhesives with bulk flowable composite was measured with Raman spectroscopy by calculating the ratio of (C=C)/(C–C) absorbance intensities (percentage of unreacted double bonds) before and after polymerization as shown in equation:

DC = [1 − (C_aliphatic_/C_aromatic_)/(U_aliphatic_/U_aromatic_)] × 100%

where C_aliphatic_ is the absorption peak at 1638 cm^−1^ of the polymerized resin, C_aromatic_ is the absorption peak at 1607 cm^−1^ of the polymerized resin, U_aliphatic_ is the absorption peak at 1638 cm^−1^ of the unpolymerized resin, and U_aromatic_ is the absorption peak at 1607 cm^−1^ of the unpolymerized resin. All experiments were performed in triplicates to calculate variations for a standard deviation.

After placement of 15 µL of adhesive inside a well, a custom made cylindrical stainless steel split-mold with an aperture diameter of 3 mm and depth of 6 mm was used to add 2 and 4 mm of bulk fill composites. The samples were cured at a relative humidity of 60% ± 15% and a room temperature of 21 °C ± 1 °C. Using a Raman spectroscope equipped with a Leica microscope and lenses (JY LabRam HR 800; Horiba Jobin Yvon, France) with curve-fitting Raman software (Labspec 5), Raman spectroscopy was performed and carbon–carbon double bond was measured at a resolution of 4 cm^−1^. The excitation parameters at zero calibration included 785 nm wavelength with argon ion 514.5 nm laser (spectral resolution of 1.6 cm^−1^) and power <500 µW at 100× objective with a superior signal/noise ratio, at the center of each specimen to generate one spectrum by holding on a universal holder. Six frames of 30 s exposures were recorded on an automated x-y-z axis-positioning stage and later subjected to system background removal, spectral analysis, and dark count correction with intensity normalized. All spectra were recalibrated to the amplitude of the spectrums presumed to also contribute from the bacterial colonies normalized to the peaks 1450 cm^−1^ (CH_2_ deformations). The specimens were visually identified using objective lens for obtaining Raman signals and precision of chemical data at different locations at room temperature and dark ambience to avoid any spikes originating from ambient light.

### 2.8. Confocal Microscopy

Prior to adhesive application, both adhesive systems were doped with 0.05 wt% Rhodamine-B (Sigma-Aldrich, St. Louis, MO, USA). Further ten sound human molars were used and restored as previously mentioned and divided into groups (*n* = 5). Two to three slabs from each tooth (*n* = 3; *n =* 15 per group) were obtained by sectioning perpendicular to the bonded surface for exposing the adhesive interface and wet polished using (600 to 2500 grit) SiC papers (Carbimet; Buehler, Lake Bluff, IL, USA), and rinsed with distilled water for 10 min under ultrasonic agitation. After gentle blotting, the bonded interfaces were analyzed using dye assisted confocal microscopy evaluation (SP5 Leica, Heidelberg, Germany) equipped with a 20× oil immersion lenses. The ultramorphology evaluation (resin-diffusion) was executed using Rhodamine excitation laser (540 nm). The confocal images were obtained with a 1 μm z-step to optically section the specimens to a depth up to 12–10 μm below the surface.

### 2.9. Statistical Analysis

The bond strength data was analyzed as means ± standard deviations using two-way ANOVA (SigmaStat Version 20, SPSS, Chicago, IL, USA). Values of the bond strength were normally distributed (Shapiro–Wilk test) and homoscedastic (modified Levene test). Utilising Tukey’s test, post-hoc multiple comparisons were performed. Weighted Kappa (κ_w_) was evaluated using inter-observer reliability and reproducibility for nanoleakage evaluation. All nanoleakage values were ordinal data and notable differences between the MI and MB groups were tested using the Cochran–Mantel–Haenszel (CMH) method (*p* < 0.05). One-way ANOVA was used for analysis of nano-hardness and degree of conversion. A significant level of *p* < 0.05 was used. Contraction gap analysis was performed using one-way ANOVA with Student–Newman–Keuls test for multiple comparisons.

## 3. Results

### 3.1. Microtensile Bond Testing

**Figure 2:**
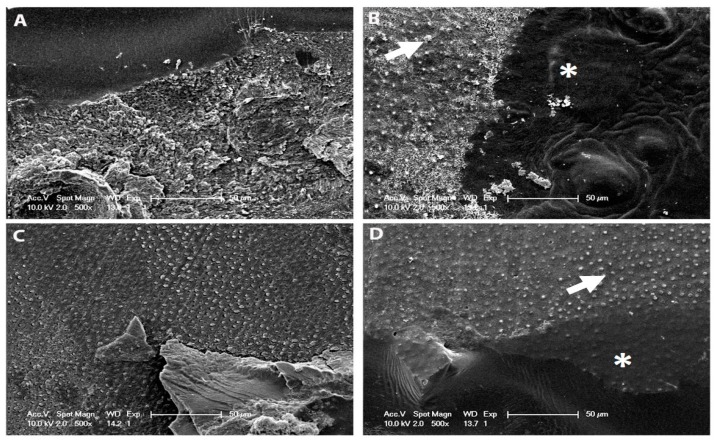
Scanning electron microscope (SEM) micrographs of dentine side of fractured beams following application of bulk-fill flow by manual blend technique. (**A**) MB Optibond; (**B**) MB Prime & Bond Universal; (**C**) MB2 Optibond; (**D**) MB2 Prime & Bond Universal specimens. Spherical globules (arrowheads) were preferentially found at the orifices of dentinal tubules in all groups except for MI Optibond FL. Evidence of bulk-fill/bonding agent (*) seen with numerous fractured resin tags (arrowheads) can be seen at the base of the hybrid layer.

The results for microtensile bond strength for each group are summarized in Table 3. Two-way analyses of variance (ANOVA) showed that the factors “storage (*p* < 0.05, F = 6.3)” and the “application (*p* < 0.05, F = 30.11)” had a significant impact on the bond strength to dentine. The interaction between “storage” and “application” (*p* < 0.001, F = 6.22) was also significant. The change in bond strength after thermocycling appeared independent of the application method and the adhesive used. The bond strength of MB2 group were significantly higher than the control and the MB4 group (*p* < 0.05). However, for the Prime & Bond Universal, the bond strengths of MB2 and MB4 groups were significantly lower than the control group (*p* < 0.05). The bond strengths of the control, MB2, and MB4 groups of Optibond FL adhesive dropped significantly after thermocycling when compared to baseline (*p* < 0.05). The bond strength of the MB2 group was significantly higher than the control and MB4 groups. No significant difference in bond strength was found between the control and MB4 groups after thermocycling (*p* > 0.05). Conversely, the bond strength of the control, MB2, and MB4 groups of Prime & Bond Universal after thermocycling was significantly lower than baseline (*p* < 0.05). After thermocycling, the bond strength of the control group was the highest, followed by MB2 and then MB4. The failure modes determined for all test specimens showed predominant mixed failures. Nevertheless, a clear prevalence of adhesive failures was observed for the MB2 and MB4 groups at baseline for both adhesives (Table 4). According to Table 4, the predominant failure mode of MB2 and MB4 at baseline is mixed failure (Figure 2).

### 3.2. Nanoleakage Testing

The weighted kappa for inter-examiner reliability beat the 0.76 cut off with a mean of 0.88; indicating excellent reproducibility. The mean weighted kappa (*k*_w_) for inter-examiner reproducibility was 0.90. Table 5 provides the distribution of nanoleakage scores for all groups of both adhesives at baseline and thermocycled aged specimens. For Optibond FL, no prove of significant difference was identified in nanoleakage score between baseline and 12 months in both the control and MB4 groups (*p* > 0.05), while significant difference in the nanoleakage score was observed in the MB2 group (*p* < 0.05). For Prime & Bond Universal, no significant difference in nanoleakage score was found between baseline and 12 months in the MB4 group (*p* > 0.05), while significant difference was evident in nanoleakage scores between the baseline and at 12 months for both the control and MB2 groups (*p* < 0.01).

### 3.3. Confocal Microscopy

**Figure 3:**
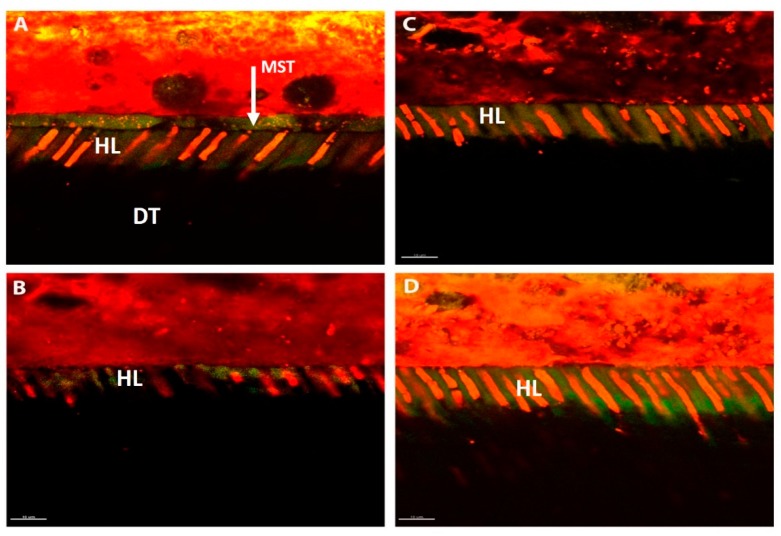
Confocal micrograph showing the hybrid layer (HL) and adhesive/bulk-fill resin penetrating dentin (DT). The width of the resin hybrid layer, thickness and length of resin tags, and the area of the adhesive resin within the dentin were measured over a 100 µm length of the axial wall. Bar represents 10 µm. (**A**) MI Optibond FL; (**B**) MI Prime & Bond Universal; (**C**) MB2 Optibond FL; (**D**) MB2 Prime & Bond Universal.

Confocal images of the resin–dentine interfaces attained in representative experimental groups (Figure 3) demonstrate the ultramorphology of the control and MB2 groups in both Optibond FL and Prime & Bond Universal adhesive showing resin hybridization in sound dentine. A rhodamine B-labeled hybrid layer and resin penetration through the porous resin–dentine interface was observed. For specimens from the MB2 Optibond FL (Figure 3C) and Prime & Bond Universal (Figure 3D), the resin was able to diffuse through the porous demineralized dentine creating a reasonable hybrid layer. The dentine interface presented funneled-shaped dentinal tubules more pronounced in the MB2 Prime & Bond Universal groups (Figure 3D).

### 3.4. SEM Resin–Dentine Interface

**Figure 4:**
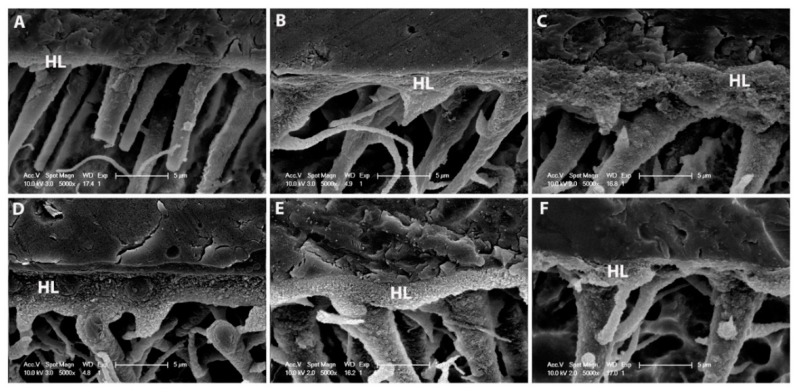
Representative SEM images of the resin–dentin interfaces bonded with manual blend technique. (**A**) Secondary electron image of MI Optibond FL. An authentic hybrid layer with 5–6-μm-thickness was observed with funnel-shaped resin tags with lateral branches; (**B**) back-scattering image of MI Prime & Bond Universal specimen; (**C**) back-scattering image of MB2 Optibond FL specimen along with (**D**) MB2 Prime & Bond Universal; (**E**) MB4 Optibond FL; (**F**) MB4 Prime & Bond Universal. (HL) Hybrid layer.

No differences were observed in the number and length of resin tags among the adhesive systems in all groups. The MB2 subgroups in both adhesive systems showed adequate hybrid layer formation at the time of analysis (Figure 4).

### 3.5. Shrinkage and Gap Evaluation

Mean and SD values for shrinkage and gap measurements after placement of the restorative materials after 24 h are shown in Table 6. There were significant differences in the width of the contraction gap among the specimens tested. The shrinkage gap was significantly reduced in the specimens from the MB2 group of both adhesive systems. About 70% of the specimens from the MB4 group of Optibond FL had a cohesive separation after recording measurements (*p* < 0.05). The specimens from the MB4 group of Prime & Bond Universal showed both mixed and interfacial separation.

### 3.6. Degree of Conversion and Micro-Raman depth Penetration

The results of DC for the different groups 30 min after light activation are depicted in Table 3. No significant difference in DC was found between the MB2 and MI groups in both adhesives (*p* > 0.05). The specimens from the MB4 group of Prime & Bond Universal exhibited the lowest DC after photo-activation, as a function of position across the resin/dentine interface. The Raman bands scanned across the resin–dentine interface at 960 cm^−1^ (P–O peak) and 1450 cm^−1^ (C–H peak) are assigned to phosphate vibrations of hydroxyapatite and the C–H alkyl group in the respective line maps. The CH band had gradually decreased for all groups across the region recorded. The depths achieved for the specimens are as follows: MI/Opti, 8.9 µm; MB2/Opti, 13.1 µm; MB4/Opti, 8.0 µm; MI/P&B, 8.0 µm; MB2/P&B, 13.6 µm; and MB4/P&B, 6.3 µm. This gradually decreased in the C–H band and reached a plateau, especially for specimens extending beyond 8 µm. The micro-Raman data from the present study confirmed a complex interaction between the resin and the resin–dentin specimens.

### 3.7. Nanoindentation

**Figure 5:**
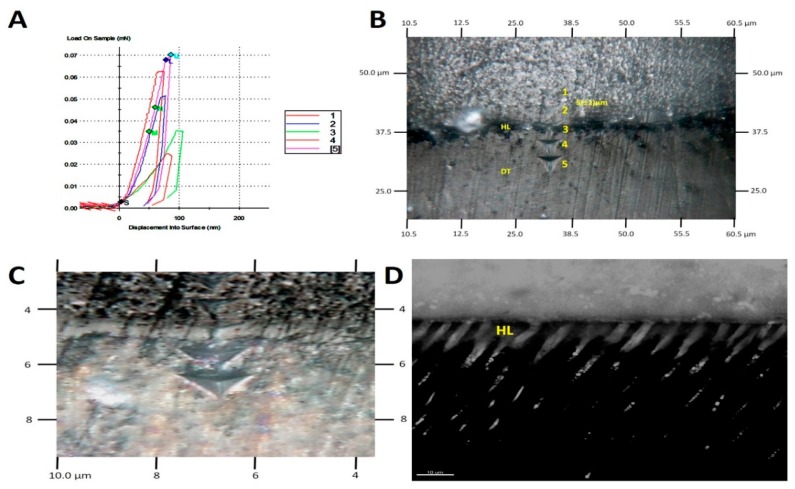
(**A**) Force deflection curves from nanoindentation of resin dentine specimens; (**B**) SEM image showing five indentations performed in straight lines along the resin dentine interface; (**C**) magnified image of nanoindentation; (**D**) fluorescence single projection of a MB2 resin dentine bonded interface showing an adequate hybrid layer along the resin tags.

Mean and standard deviations of nano-hardness (Hi) along the resin–dentine hybrid layer are shown in Table 1. The resin–dentine hybrid layer created by the MB2 technique in Optibond FL adhesive attained in wet condition showed significantly higher values (*p* < 0.05) at the equivalent interface positions. No statistical differences were observed between the Optibond FL MB4, MI/MB4 Prime & Bond Universal groups. The AFM imaging performed in dry conditions showed that the hybrid layer originated by using the MB2 technique in both adhesives presented collapsing phenomenon within the interface (Figure 5).

## 4. Discussion

Nowadays, low shrinkage bulk-fill resins are widely used clinically, in single increment application of up to 4–5 mm [36]. These resins initially had a flowable consistency for use as base materials or liners, which are then complemented with a final layer of a conventional composite resin. In SDR^®^ Flow (DENTSPLY, Konstanz, Germany), urethane-based methacrylate resins were incorporated with photo-initiators as a backbone to facilitate radical polymerization process and consequently provided greater flexibility and dissipation of stress during actual polymerisation [37]. SDR flow had shown a greater depth of cure than flowable resins, conventional flowable composites, and restorable bulk-fill composites [38]. Furthermore, the hardness of the new SDR Flow+ was improved according to the manufacture claims. In this present study we have proposed a step-by-step procedure to restore permanent molars with a “manual blend technique”. The technique of choice in this case includes mixing of a bulk-fill flowable composite resin restorative material with un-cured dentine bonding agent, then completing the rest of the cavity by using a conventional paste composite resin restorative material. The resin results used has superior mechanical properties and it may be justified to incorporate the bonding layer using the new method to protect the material from potential wear [39]. According to Hirata et al., high density bulk-fill used in a single increment up to 4 mm may be difficult to place. This may also have detrimental effects on the final aesthetic result [40]. However, adding an extra coat of filled, hydrophobic bulk-fill flowable composite resin material to the uncured adhesive may reduce the hydrophilicity and water sorption of simplified dentine bonding agents, stabilizing the hybrid layer over time [28]. The diffusion process of the adhesive system inside the demineralized area would have remained the same as that in the traditional technique as it may depend on each adhesive type and composition. The concept initiates with the key parameter that bulk fill composites, when placed in deep cavities, have improved depth of cure [41]. The authors showed lower nanoleakage scores with 2 mm bulk fill in the two different layering technique. This procedure prevents the authors from considering the traditional method of curing adhesive and bulk fill separately as restorations had better marginal adaptation. The resin material seemed optimally cured, and hence optimum mechanical properties were achieved.

In this study, there was a significantly higher microtensile bond strength for the MB2 group of Optibond FL adhesive systems as compared to the control. Conversely, the bond strength for the MB2 group of Prime & Bond Universal adhesive was significantly reduced. The isopropanol alcohol solvent present in Prime & Bond Universal adhesive is known to induce a change in the axial relationship between collagen molecules along the fibril axis, decreasing intermolecular packing [42]. This may be due to disruption of the amino acid bonding along the collagen triple helix, which subsequently induces rearrangement of intra and intermolecular bonding [28]. Shrinkage of a thinner layer of bulk-fill (2 mm) resin with bonding agent in the MB technique may also contribute to a lower C-factor and reduce the stress associated with the polymerization shrinkage. All these contribute to an increased adhesion of the composite to dentine [43]. Traditionally, the filling material is placed into the cavity in one bulk application; it gets in contact with five walls and only one free surface, hence in such, the C-factor is maximal [44,45]. The authors also speculate, that there may have been a diffusion of the acidic primer inside demineralized dentine with the adhesive. The dimethacrylates have the ability to cluster together before polymerization to create hydrophobic domains, obtaining an even distribution of hydrophilic and hydrophobic components. Phase separation can create water-trees and uneven, weaker interfaces, which will affect the durability of the bonded interfaces [19]. The failure mode was predominantly mixed failures for both MB2 groups of the different adhesive systems. No remarkable differences were observed on visual comparison and on contraction gap analysis.

Contraction stress and gap formations can be influenced by many factors such as the kinetics of polymerization, resin matrix composition, class of resins, and filler content. This will dictate volumetric shrinkage happening within the polymer composite [46]. The SDR Flow+ used in this study has silanated barium and strontium glass fillers (Dentsply data), which may be speculated to have shown lower contraction stress and produced smaller contraction gaps than the MB4 group in our study. Another possible reason for smaller gap formation within the MB2 groups is that the 2 mm bulk-fill flowable rubbed on uncured bonding agent may lead to slow chemical curing, reducing the gap formation. Differences in bond strength between composites can be attributed to differences in shrinkage stress. Shrinkage stress is not a material’s property but is inherent to the compliance and C-factor [47]. The bulk-fill also demonstrates greater depth of cure, which had far ranging effect when applied in 2 mm increments and rubbed onto the uncured bonding agent. The developing polymer’s viscosity is still low; therefore, shrinkage stress is compensated by the plastic flow of the bulk-fill flowable composites and slow polymerization [48]. For this particular reason, the internal stresses within the material are relaxed and it turned out that the MB2 group showed superior mechanical properties (Optibond FL adhesive) and lesser gap formation overall. The specimens treated with 4 mm bulk-fill flowable composite may have a delayed gelation point. Most manufacturers have recommended successful bulk resins do not exceed 4 mm in depth [49,50]. However, our results suggested a co-cure approach with 2 mm bulk fill flowable composite as the bond strengths were comparable. These findings agree with the results observed in the study which showed optimum hybrid layer formation as seen in the SEM and confocal images. The multifunctional monomers formed adequate tag formation and hybrid layer in the MB groups of both adhesive systems. This also justified the high nano-hardness (Hi) and hybrid layer formation observed in this study. The use of material has had a positive impact, as the amount of polymerization shrinkage is a factor of the type and composition of composite resin used rather than any other factors of light curing [51]. With respect to these findings, the null hypotheses that new application method of bulk-fill flowable composite resin materials has no effect on the micro-tensile bond strengths and on contraction gaps after polymerization has to be rejected. These findings are a clear indication that the technique with 2 mm bulk fill flowable may have the same efficiency in obtaining adequate depth of cure.

This study showed that with an etch-and-rinse adhesive modified technique, it is possible to successfully work with a 2-mm bulk layer. Concurrently, the nano-hardness values and DOC of the specimens from the MB2 group of Optibond FL showed significant improvement compared to the MB4 and control groups. In addition, the specimens from the MB2 group showed statistical difference with control MI specimens for both adhesive groups, with high numerical values seen for DOC. The barium glass present inside Optibond FL may have had a dual effect with the filler content of bulk-fill resin in increasing the mechanical properties. The slow polymerization of the resin is another mechanism that compensates stresses in composite resins; it increases the resin’s flow capacity as mentioned previously.

The Raman peaks at 1450 cm^−1^ correspond to the CH_2_CH_3_ present in most monomers and are indicative of resin penetration within the dentine specimens. The present study aimed at analyzing changes within the known Raman peak positions along the depth of the resin/dentine interface. The specimens from the MB2 group of both adhesives showed higher amounts of penetration compared to other specimens. The penetration depths for the different specimens seemed to be dependent on the 2 mm bulk-fill resin application. There was a likely reason that the photo-initiators of the dentine–adhesive system in absorbing the blue light, could be affected by the thickness of the bulk resin used. Subsequently, with an increase in bulk-fill resin up to 4 mm in both groups, there was no substantial increase in the penetration depths. Therefore, the 2 mm bulk-fill resin may justify and explain the adequate depth of cure. Accordingly, the null hypothesis can be rejected because the MB2 technique affected the resin penetration of the adhesive. However, further studies are required to further evaluate the changes happening within the hybrid layer as well as, their performance in long-term in vivo studies.

## 5. Clinical Significance

Clinicians may find the procedure easy to perform as it can be executed quickly. Advantages of the presented technique include a greater stability of the hybrid layer with no real change in the clinical restorative time. Moreover, fewer composite layers may be required as compared to the classical layering composite technique in the posterior teeth.

## 6. Conclusions

Present study suggests that the manual blend technique might have a positive effect on bond strengths and longevity of etch-and-rinse adhesives to dentine.

## Figures and Tables

**Table 1:** Chemical composition and components of Optibond FL, Kerr and Prime & Bond Universal, Dentsply adhesive systems.

Dental Bonding Agent (DBA)		
Brand Name, Manufacturer	Optibond FL, Kerr	Prime & Bond Universal
Resin monomers	Primer: HEMA, GPDM, PAMM Bonding: TEGDMA, UDMA, GPDM, HEMA, bis-GMA	PENTA (dipentaerythritol pentacrylate phosphate), 10-MDP (10-methacryloyloxydecyl dihydrogen phosphate), Active Guard^TM^ Technology crosslinker
Initiator system	CQ/tertiary amine	CQ/tertiary amine
Filler load	48 w/o	Unfilled
Filler size	0.6 μm	-
Filler type	Barium glass	-
Solvent type	Ethanol, water	Isopropanol, water
Solvent content	20–30 w/o	10–24.5% Isopropanol, 5–24.5% water
Others	Fluoride	-
Delivery systems	bottle	bottle

**Table 2:** Chemical composition and components of Ceram.X One and SDR Flow+ composite resins.

Composite Resin Restorative Material		
Brand name, manufacturer	CERAM.X ONE, Dentsply	SureFil SDR flow+, Dentsply
Resin monomers	Poly-urethanemethacrylate, bis-EMA, TEGDMA	Modified urethane dimethacrylate resin, ethoxylated bisphenol-A dimethacrylate (EBPADMA), triethyleneglycol dimethacrylate (TEGDMA)
Initiator/accelerator system	Camphorquinone (CQ)/butylated hydroxyl toluene (BHT)	Camphorquinone (CQ)/butylated hydroxyl toluene (BHT)
Filler load	77–79 w/o (59–61 v/o)	47.3 v/o
Filler size	Organic fillers ≈ 15 μm, non-agglomerated inorganic fillers ≈ 0.6 μm	Inorganic filler from 0.02–10 μm
Filler type	Barium glass and ytterbium fluoride fillers	Silanated barium and strontium glass fillers, ytterbium fluoride fillers, Nano fillers
Filler shape	Spherical, spray-granulation production method	Unagglomerated (barium and strontium glass fillers), irregularly shaped

**Table 3:** Values of means ± standard deviations, presented in MPa, showing microtensile bond strength after 24 h and following thermocycle ageing in artificial saliva. Uppercase letters/lowercase letters constitute differences between each row while symbols (α, β, γ and ∞) represent differences between the columns. Symbols (†, ‡, ‡‡) indicate differences in degree of conversion between different adhesives.

Adhesives	Groups	24 h	Thermocycling 10,000	DOC % (30 min after light activation)	Nano Hardness (Hi) GPa
Optibond FL, Kerr	MI	32.1 ± 4.4 A α	29.9 ± 3.3 a β	89.22 ± 1.9 ‡‡	5.9 ± 0.8 A
	MB2	35.5 ± 5.2 C β	33.3 ± 7.1 b γ	91.43 ± 3.3 ‡‡	9.8 ± 1.1 B
	MB4	30.1 ± 8.0 AB α	28.7 ± 6.3 a β	78.66 ± 1.8 ‡	3.9 ± 2.3 C
Prime & Bond Universal	MI	36.4 ± 5.5.5 B β	31.7 ± 8.0 b γ	80.66 ± 3.1 ‡	4.3 ± 0.9 C
	MB2	33.1±6.1 A α	29.8 ± 6.6 a β	82.11 ± 1.5 ‡	6.4 ± 2.2 BC
	MB4	27.3 ± 4.9 D ∞	24.1.2 ± 2.3 c ∞	67.61 ± 2.5 †	3.8 ± 2.1 C

**Table 4:** Showing the percentage distribution in the mode of failure after 24 h and following thermocycling of aging in artificial saliva. Manner of failure presented: A, at adhesive; CD, cohesive failure in dentine; CC, cohesive failure in resin composite; and M, mixed failure.

Sub Groups	Failure Mode	Optibond FL, Kerr		Prime & Bond Universal^TM^	
		24 h	Thermocycling 10,000	24 h	Thermocycling 10,000
**MI**	A	29	39	14	23
	M	45	33	44	51
	CD	11	8	20	7
	CC	15	20	22	19
**MB2**	A	19	23	32	10
	M	51	47	60	45
	CD	15	8	4	18
	CC	15	22	4	27
**MB4**	A	21	31	29	27
	M	40	39	38	45
	CD	18	6	12	11
	CC	21	24	4	17

**Table 5:** Outline of nanoleakage scores for the five treatment groups of both adhesives at baseline and after thermocycling. The extent of interfacial nanoleakage presented as 0: 0%; 1: <25%; 2: 25–50%; 3: 50–75%; 4: >75%. Cochran–Mantel–Haenszel test (2 × 2 × 5).

Optibond FL, Kerr	Sub Groups	Nanoleakage Score					
		**0**	**1**	**2**	**3**	**4**	*p*-value
**Baseline**	MI	12	15	17	2	6	0.066
	MB2	6	8	12	12	3	0.03
	MB4	8	9	21	14	5	0.512
**Thermocycled**	MI	1	0	16	16	6	
	MB2	3	9	28	10	1	
	MB4	0	11	24	5	6	
**Prime & Bond Universal**	**Sub Groups**	**Nanoleakage Score**					
		**0**	**1**	**2**	**3**	**4**	*p*-value
**Baseline**	M1	4	18	7	9	6	0.000
	MB2	3	10	6	11	7	0.001
	MB4	0	0	16	10	11	0.222
**Thermocycled**	M1	0	0	12	9	16	
	MB2	5	0	9	17	5	
	MB4	0	0	22	3	4	

**Table 6:** Contraction gap width (µm) as a function of location.

**Optibond FL**	**Sub Groups**	**Estimated Mean**	***p*-value**
	MI	4.11 ± 1.2	0.23
	MB2	0.87 ± 0.09	0.02
	MB4	2.12 ± 0.9	0.31
**Prime & Bond Universal**	MI	6.11 ± 2.1	0.77
	MB2	2.43 ± 0.81	0.04
	MB4	2.12 ± 0.6	0.9
